# Signaling via the FLP-14/FRPR-19 neuropeptide pathway sustains nociceptive response to repeated noxious stimuli in *C*. *elegans*

**DOI:** 10.1371/journal.pgen.1009880

**Published:** 2021-11-08

**Authors:** Filipe Marques, Laurent Falquet, Elke Vandewyer, Isabel Beets, Dominique A. Glauser

**Affiliations:** 1 Department of Biology, University of Fribourg, Fribourg, Switzerland; 2 Swiss Institute of Bioinformatics, Lausanne, Switzerland; 3 Neural Signaling and Circuit Plasticity Group, Department of Biology, KU Leuven, Leuven, Belgium; University of California San Francisco, UNITED STATES

## Abstract

In order to thrive in constantly changing environments, animals must adaptively respond to threatening events. Noxious stimuli are not only processed according to their absolute intensity, but also to their context. Adaptation processes can cause animals to habituate at different rates and degrees in response to permanent or repeated stimuli. Here, we used a forward genetic approach in *Caenorhabditis elegans* to identify a neuropeptidergic pathway, essential to prevent fast habituation and maintain robust withdrawal responses to repeated noxious stimuli. This pathway involves the FRPR-19A and FRPR-19B G-protein coupled receptor isoforms produced from the *frpr-19* gene by alternative splicing. Loss or overexpression of each or both isoforms can impair withdrawal responses caused by the optogenetic activation of the polymodal FLP nociceptor neuron. Furthermore, we identified FLP-8 and FLP-14 as FRPR-19 ligands *in vitro*. *flp-14*, but not *flp-8*, was essential to promote withdrawal response and is part of the same genetic pathway as *frpr-19 in vivo*. Expression and cell-specific rescue analyses suggest that FRPR-19 acts both in the FLP nociceptive neurons and downstream interneurons, whereas FLP-14 acts from interneurons. Importantly, genetic impairment of the FLP-14/FRPR-19 pathway accelerated the habituation to repeated FLP-specific optogenetic activation, as well as to repeated noxious heat and harsh touch stimuli. Collectively, our data suggest that well-adjusted neuromodulation via the FLP-14/FRPR-19 pathway contributes to promote nociceptive signals in *C*. *elegans* and counteracts habituation processes that otherwise tend to rapidly reduce aversive responses to repeated noxious stimuli.

## Author summary

We all rapidly habituate to persistent or repeated innocuous sensory stimuli, for example when we go swimming and quickly accustom to cool water. In contrast, plasticity mechanisms in the nociceptive pathways tend to produce habituation for painful sensations only on longer time scales. Rapidly ignoring persistent innocuous stimuli, but remaining sensitive to potentially damaging stimuli is hence a strategy universally applied in animals. However, little is known about the molecular mechanisms acting to prevent rapid habituation in nociceptive pathways, despite understanding these mechanisms may provide important clues for developing new therapeutic approaches in pain management. Here, we identify a neuropeptide-based positive feedback signaling in a nociceptive pathway, which is essential to maintain its activity in a paradigm of repeated noxious stimulations.

## Introduction

In order to survive, animals have developed strategies to avoid harmful situations. Noxious stimuli are detected by specialized sensory neurons, called nociceptors, who transmit the information downstream in the nervous system for additional processing and the elicitation of aversive behaviors, which ultimately help limiting damages [1,2]. In human, the activation of nociceptive pathways results in pain sensation, an unpleasant sensory experience. Whereas physiological pain represents a useful warning signal, many maladaptive forms of pain, such as neuropathic and inflammatory pain, significantly decrease well-being and may become a major burden in patients, notably when these conditions become chronic [3]. Hence, considerable research efforts are dedicated to understand the molecular and cellular processes modulating nociception in normal and pathological conditions [4–8]. The activity of nociceptive pathways is not fixed, but rather subject to plasticity processes [9,10]. Nociceptive plasticity includes both pro-nociceptive and anti-nociceptive mechanisms, which may occur in different contexts. For example, repeated or long-lasting noxious stimulations may cause activity-dependent hypersensitization or habituation. Habituation can be defined as a behavioral response decrement caused by repeated stimulations [11]. Interestingly, nociceptive habituation was shown to be reduced in patients with different chronic pain conditions [12,13]. Nociceptive plasticity phenomena are deemed relevant to understand the etiology of chronic pain in human [14,15].

Nociceptive pathways are modulated by a wide range of molecular signals, such as neuropeptides [16]. Neuropeptides form a heterogeneous family of intercellular communication molecules, which activate G protein-coupled receptors (GPCRs) at the membrane of target cells and produce pleiotropic effects. In sensory systems, neuropeptide signaling is involved in the context-dependent modulation of neuron function, a well-conserved role reported in a variety of organisms from human to invertebrates [17–19]. Various neuropeptides have been involved in the transmission, modulation and perception of all types of pain (physiological, neuropathic and inflammatory) [20]. Neuropeptides can have pro-nociceptive or anti-nociceptive actions, and sometimes both pro- and anti-nociceptive actions depending on the context and on their place of action in the nervous system. This is the case for example for Neuropeptide Y and for the FRMF amide family of neuropeptides [21–23]. In addition, neuropeptides can have different ranges of action, being involved in both synaptic and non-synaptic transmission and working in an autocrine, paracrine or even endocrine manner [24]. Because of the widespread expression of neuropeptides and their receptors, and their functional versatility, the modulatory action of neuropeptide signaling is particularly challenging to define *in vivo*. Research about the molecular signaling controlling nociceptive processing and plasticity is hindered in mammals by ethical concerns, by the size and the anatomical complexity of their nervous system, and by the relative slowness of genetic approaches. Because they circumvent all these limitations, and because their nociceptive molecular pathways are remarkably conserved, invertebrates (such as *Caenorhabditis elegans*) have recently emerged as efficient complementary research models.

Neurogenetic studies with *C*. *elegans* have been very successful in dissecting the molecular and cellular mechanisms involved in sensory-behavior connections [25–27] and in modeling human diseases, including neurological conditions [28–30]. With the availability of efficient genetic methods and a fully mapped nervous system, *C*. *elegans* has thus become an outstanding model, not only for identifying the molecular/genetic factors involved, but also, and even more remarkably, to bridge our understanding of mechanisms across the molecular, cellular, neural circuit, and behavioral levels. *C*. *elegans* expresses a large set of neuropeptides and receptors, with a conserved neuromodulatory role, including as modulator of nociceptive pathways [17,31–33]. Worms produce avoidance behaviors in response to noxious stimuli, such as harsh touch and noxious heat [34,35], offering interesting experimental paradigms to study nociceptive function.

When pocked on the head or exposed to noxious heat, worms produce a robust innate reversal response, consisting in a period of backward locomotion over a distance that may vary according to the stimulus intensity and the previous sensory experience [36,37]. Noxious heat-evoked reversal response involves AFD, AWC and FLP thermosensitive neurons [38–41]. Responses to mechanical stimuli rely on several head mechanosensitive neurons including ALM, AVM, ADE, CEP, OLQ, ASH and FLP [42]. Like for other types of sensory-behavior in *C*. *elegans*, the nociceptive pathway seems to form a functionally degenerate circuit, with several parallel pathways converging on the elicitation of reversal behavior [43,44]. This partial functional redundancy across multiple neural pathways is probably a key to the robustness and flexibility of essential, potentially life-saving defensive behaviors. Involved in the response to both heat and touch stimuli, FLP is a polymodal nociceptor with a multi-dendritic anatomy, innervating the whole animal head [45]. FLP is presynaptic to several reversal-promoting interneurons: AVA, AVD, AVE and AIB. An optogenetic activation of FLP is sufficient to trigger reversal [46], a response relying on intact glutamate transmission [47]. Harsh touch-evoked and FLP activation-evoked reversal responses habituate very slowly to repeated stimulations. It takes more than 5 stimuli to start seeing signs of habituation and the effect remains modest (~20% reduction) after 15 to 20 stimuli. In comparison, habituation to innocuous stimuli is markedly faster. E.g., habituation seen in the gentle-touch pathway, either via repeated gentle touch or repeated optogenetic activation cause a 50–80% response reduction after 15 to 20 stimuli [46,48]. The mechanistic reason why responses to noxious stimuli habituate much less than responses to innocuous stimuli remains unclear.

Recently, we designed a forward genetic approach to screen for mutations impairing the reversal behaviors caused by the selective optogenetic activation of FLP [47]. Activating a single sensory neuron presents two main advantages: first it circumvents limitations associated with the high robustness of a degenerate neural circuit, whose function is intrinsically difficult to disrupt. Second, it directly highlights the relevant neural pathway in which identified mutations are most likely to act. Mutants were screened with a five-trial stimulation protocol, which in wild type elicits a robust response with no sign of habituation. With this procedure, we could recover mutants with a global responsiveness decrease, as well as mutants with faster habituation. Here, we report the identification of a nonsense mutation in the neuropeptide receptor-encoding *frpr-19* gene and its validation as the cause of impaired reversal behavior. By using a variety of genome editing and overexpression approaches, we show that the normal expression of both FRPR-19A and FRPR-19B isoforms is essential to maintain the responsiveness in the FLP neuron pathway. We furthermore deorphanize FRPR-19, showing that it is a receptor for FLP-8 and FLP-14, the latter one being essential to regulate reversal behavior. Moreover, we identify the place of action of *frpr-19* and *flp-14* to suggest a circuitry model for the pathway, involving a feedback on FLP. Finally, we demonstrate the importance of the FLP-14/FRPR-19 pathway for preventing habituation to natural stimuli sensed by FLP, namely noxious heat and harsh touch. Collectively, our results highlight the importance of a single neuropeptide targeting both sensory neurons and interneurons in the modulation of nociceptive habituation.

## Results

### A nonsense mutation in *frpr-19* impairs FLP neuron-dependent reversals

**Fig 1:**
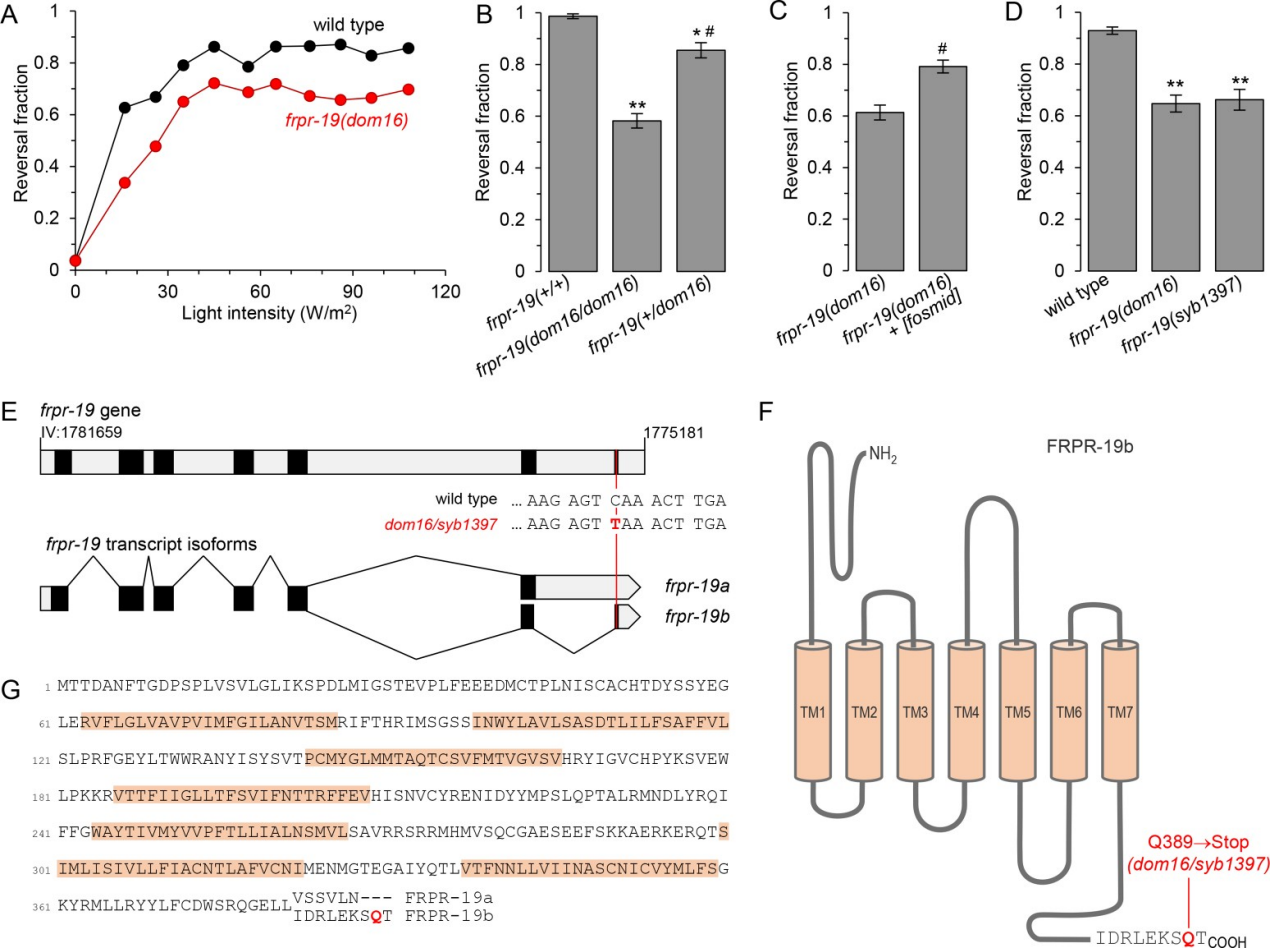
A nonsense mutation in *frpr-19* reduces FLP neuron-dependent reversals. (A) Light intensity-response curves of wild type and *frpr-19(dom16)* mutants. All animals contained the *domIs272[FLP*::*CoChR]* transgene enabling the optogenetic activation of FLP. Reversal fraction scored over 5 trials per animals. *n* = 100 animals. (B, C, D) Fraction of FLP optogenetic stimuli producing a reversal response as in A, with 61 W/m^2^ light stimuli, and showing the slight semi-dominance of the *frpr-19(dom16)* allele (B), the rescue effect with a WRM0623dC10 (*frpr-19-*containing) fosmid (C), and a phenocopy effect by an *frpr-19(syb1397)* CRISPR/Cas9 engineered allele causing the same mutation as *dom16* (D). **, *p* < .01; *, *p* < .05 versus wild type control and #, *p* < .01 versus *frpr-19(dom16)* homozygotes by Bonferroni contrasts. (E) Structure of the *frpr-19* gene on the (-) strand of chromosome IV (note the genomic coordinates) and of the two transcript isoforms formed by alternative splicing in the 3’ region of the gene. Coding exons (black), introns and UTRs (grey), nucleotide change causing the nonsense mutation (red). (F) Predicted topology of FRPR-19B with the isoform-specific primary sequence highlighted. (G) Primary sequence of FRPR-19A/B, with an alignment of the divergent sequence between the two isoforms at the C-terminus.

By screening for mutants impairing the reversal response to the selective optogenetic activation of FLP nociceptor neurons, we recovered a homozygote mutant carrying the *dom16* allele. The light dose-response curve was slightly right-shifted in this mutant and the maximal reversal rate reached was lower than that in wild type (Fig 1A). Whereas our initial assessment suggested that *dom16* was recessive and we could map this mutation based on this assumption, a quantitative analysis eventually indicated a slight level of semi-dominance (Fig 1B). EMS-variant density mapping placed *dom16* on chromosome IV and we selected as primary candidate the mutation with the highest predicted impact: an A to T (Q389->Stop) nonsense mutation in the *frpr-19* gene (Fig 1E). Two transcript isoforms are reported for *frpr-19* in wormbase (WBcel235 [49]) owing to alternative splicing at the 3’ end of the gene (Fig 1E). The two *frpr-19* transcript isoforms were modeled based on RNA-seq data [50]. They encode two G protein-coupled receptors (GPCRs) diverging in their C-terminal intracellular tails: FRPR-19A and FRPR-19B (Fig 1F and 1G). We examined genomic sequences in the *Caenorhabditis* genus and found very similar putative alternative exons for *frpr-19* gene homologs (S1A Fig). The amino acid sequences in the respective predicted protein isoforms were almost identical (S1B Fig). This marked sequence conservation suggests that both isoforms might have a conserved function.

The *dom16* allele is predicted to remove the last two amino acids in the FRPR-19B isoform, but to leave the FRPR-19A sequence intact. Previous studies have shown that the very last amino acids of GPCRs are often critical for their function [51]. We used two approaches to confirm that this nonsense mutation was indeed causing the behavioral phenotype. First, we rescued the FLP-evoked reversal defect in *dom16* mutants with a fosmid containing the *frpr-19* gene and surrounding genomic sequence (Fig 1C). Second, we could recapitulate the *frpr-19(dom16)* phenotype in a *frpr-19(syb1397)* CRISPR/Cas9-engineered mutant containing the exact same nonsense mutation (Fig 1D). Collectively, these results demonstrate that a Q389 nonsense mutation in the *frpr-19* receptor gene is sufficient to impair the reversal behavior pathway downstream of FLP nociceptor neurons.

### Proper expression of both FRPR-19 isoforms is essential for FLP neuron-dependent reversals

**Fig 2:**
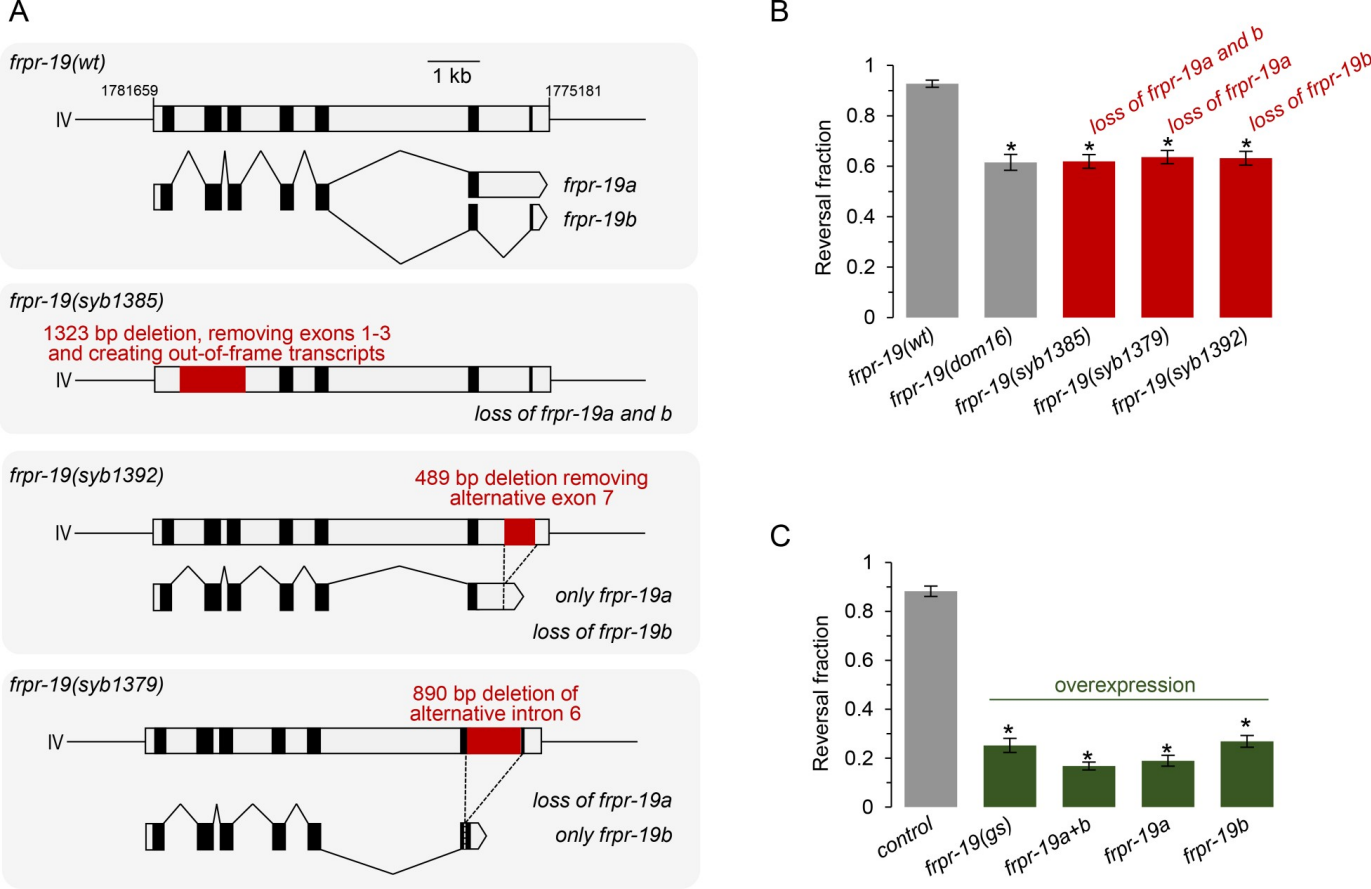
Both FRPR-19A/B loss and their overexpression impair FLP neuron-dependent reversals. (A) Schematic of wild type and engineered *frpr-19* mutations showing, for each, the genomic locus (top) and the resulting transcript (bottom). (B, C) Fraction of FLP optogenetic stimuli producing a reversal response, scored as in Fig 1, in the indicated *frpr-19* mutants (B) and in *frpr-19(wt)* animals containing overexpression transgenes as multi-copy extrachromosomal arrays (C): *frpr-19(gs)* (genomic sequence); *frpr-19a+b* (cds constructs for both a and b isoforms); *frpr-19a* (cds construct for the a isoform); *frpr-19b* (cds construct for the b isoform). *, *p* < .001 versus wild type control by Bonferroni contrasts.

The results obtained with the *frpr-19(dom16)* mutant indicate that FRPR-19 (and in particular the FRPR-19B isoform) controls the function of the neural pathway working downstream of FLP activation. However, because it is difficult to predict and, *a fortiori*, to assess the functional impact of the Q389Stop mutation *in vivo*, we pursued our analysis with three CRISPR/Cas9 engineered *frpr-19* mutants. In the first mutant (*syb1385*), we deleted 1323 bp covering the first three exons and creating out-of-frame transcripts (Fig 2A). Based on this design and on the data reported below, we consider that it represents a null allele. In the second mutant (*syb1392*), we removed the 489 bp of alternative exon 7, preventing the creation of *frpr-19b* transcript (Fig 2A). This mutant can only produce *frpr-19a*. In the third mutant (*syb1379*), we removed the 890 bp of alternative intron 6, preventing the formation of *frpr-19a* transcript (Fig 2A). This mutant can only produce *frpr-19b*. We next assessed the FLP-stimulation-evoked reversal responses in these mutants. Quite remarkably, all three mutants displayed a decreased responsiveness indistinguishable from that in *frpr-19(dom16)* mutant (Fig 2B). These results indicate that *frpr-19* is essential for FLP-evoked reversal behavior and that both *frpr-19* isoforms non-redundantly regulate FLP-dependent behavioral response.

Next, we wanted to evaluate the impact of FRPR-19 signaling upregulation. Because a dynamic equilibrium exists between GPCRs in their inactive and active forms even in the absence of agonist, the activation of intracellular G-protein signals can be triggered by GPCR overexpression, an approach recurrently used in *C*. *elegans* [52–55]. Therefore, we carried out different *frpr-19* overexpression experiments using extrachromosomal array-containing lines with multiple transgene copies. We created transgenes fusing a 3 kb *frpr-19* promoter region upstream of either a *frpr-19* genomic sequence (*frpr-19(gs)*) or the coding sequences for *frpr-19a* or *frpr-19b* isoforms. When transformed in wild type worms, all the overexpression constructs caused marked defects in FLP-evoked reversals (Fig 2C), which were even more severe than in *frpr-19* loss-of-function mutants. This was the case with the *frpr-19* genomic sequence, with only *frpr-19a* isoform, with only *frpr-19b* isoform, as well as with a combination of both *frpr-19a* and *frpr-19b* isoforms. This was also the case in a *frpr-19(syb1385)* null background (S2 Fig). Furthermore, we could see a dose-dependent effect, with a lower transgene concentration causing a milder phenotype than a higher concentration (S2 Fig). Collectively, these results demonstrate that *frpr-19* overexpression in a variety of contexts, consistently decreases FLP-evoked response.

Considering the facts that behavioral impairments are caused by single isoform perturbations and by both loss-of-function and overexpression, we conclude that the proper expression and activity levels of the two FRPR-19 receptor isoforms are essential to adjust the responsiveness in the reversal neural pathway downstream of FLP activation. The non-redundancy between the two isoforms suggests that they function in a complementary manner, either by working as separate receptors or as part of heteromeric receptors with unique properties.

### FRPR-19 acts in FLP sensory neurons as well as interneurons to modulate reversal behavior

**Fig 3:**
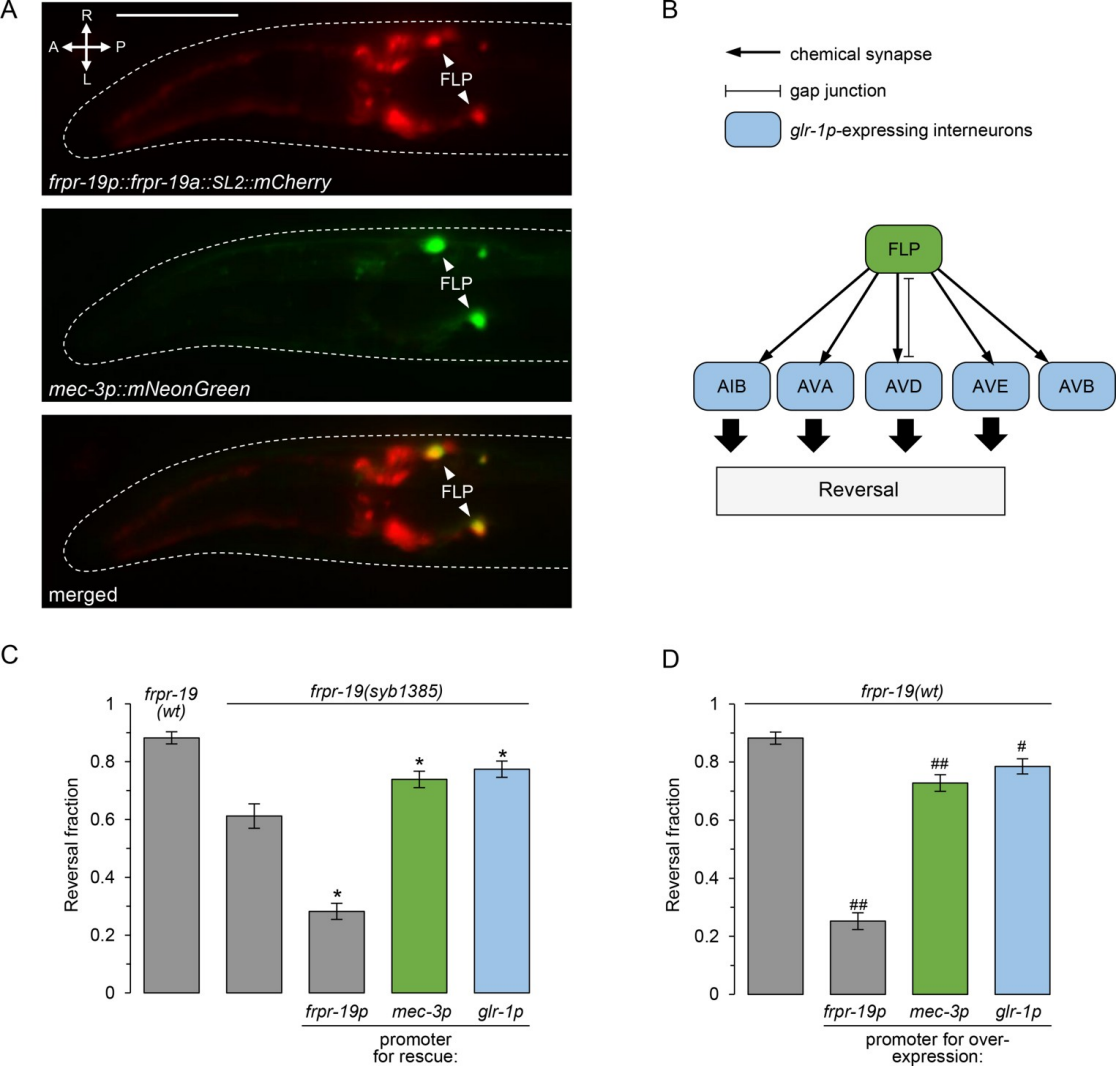
FRPR-19 acts cell autonomously in FLP neurons and downstream interneurons. (A) Epifluorescence micrography of the head of a transgenic adult, showing the expression pattern of a transcriptional reporter for the *frpr-19p* promoter (mCherry, red, top), the localization of FLP with a *mec-3p* reporter (mNeonGreen, green, middle) and a merged picture (bottom). (B) Schematic showing FLP and all its postsynaptic partners who all express *glr-1*. (C) Cell-specific rescue experiments in the *frpr-19(syb1385)* null background showing a significant rescue effect with *mec-3p* promoter driving expression in FLP and with the *glr-1* promoter driving expression in interneurons. Fraction of FLP optogenetic stimuli producing a reversal response, scored as in Fig 1. *, *p* < .01 versus *frpr-19(syb1385)* by Bonferroni contrasts. (D) Cell-specific overexpression experiments in *frpr-19(wt)* background showing a strong reversal decrease with the *frpr-19p* promoter and a partial decrease with *mec-3p* and *glr-1p* promoter. Fraction of FLP optogenetic stimuli producing a reversal response, scored as in Fig 1. ##, *p* < .01; #, *p* < .05 versus non-transgenic control by Bonferroni contrasts.

Next, we wanted to determine the place of action of the *frpr-19* gene in regulating FLP-dependent reversal behavior. Previous single-cell RNA-sequencing (scRNA-seq) analyses indicated that *frpr-19* is expressed in several neurons including FLP, at least one interneuron postsynaptic to FLP (AVD), and motor neurons, which are essential effectors in the circuit controlling forward and backward locomotion drives (VA, DA, VB, DB) [56]. The expression of a *[frpr-19p*::*frpr-19a*::*SL2mCherry]* reporter was in line with this scRNA-seq-based profile. In particular, we could confirm the expression in FLP via a co-labeling with a *[mec-3p*::*mNeonGreen]* reporter (Fig 3A).

In order to test in which neurons *frpr-19* expression is sufficient to promote FLP-dependent reversal behavior, we performed cell-specific rescue experiments using plasmids containing the *frpr-19* genomic sequence (gs), which should be able to recapitulate normal isoform splicing in the cell in which they are transcribed. Like mentioned above, even with relatively low plasmid concentrations, we could not rescue *frpr-19(syb1385)* phenotype by using the *frpr-19p* promoter and instead aggravated the phenotype. This could be due to an overexpression effect, and/or to the inability of the promoter to faithfully recapitulate normal *frpr-19* expression, potentially causing an imbalanced FRPR-19 signaling across reversal-promoting and reversal-inhibiting neurons. Nevertheless, when using the *mec-3* promoter to drive *frpr-19(gs)* expression in FLP, as well as a few other touch receptor neurons, we could see a significant rescue effect (Fig 3C). A similar rescue effect was produced with a *glr-1* promoter [57], which is expressed in all the interneurons postsynaptic to FLP (Fig 3B and 3C). The expression patterns of SL2::mCherry co-makers present in these rescue constructs was consistent with the previously reported patterns for *mec-3* and *glr-1* promoters. Whereas we cannot rule out the contribution of additional cell types, including cell types where mCherry expression is below detection threshold, these results suggest that *frpr-19* acts both in FLP and its downstream interneurons in the reversal neural pathway in order to regulate aversive behaviors.

Since *frpr-19* overexpression with *frpr-19p* can impair FLP-evoked reversal response, we next wondered if selective *frpr-19* overexpression in FLP or its downstream interneurons could produce the same effect. We therefore created transgenic lines in the *frpr-19(wt)* background containing the *[mec-3p*::*frpr-19(gs)]* or *[glr-1p*::*frpr-19(gs)]* plasmids. Both transgenes caused a significant decrease in the reversal response (Fig 3D). These defects were however much milder than those caused by the overexpression driven by *frpr-19* promoter, suggesting that the drastic phenotype in the latter case involves overactive FRPR-19 signaling in multiple neurons.

Collectively, the results of cell-specific rescue and overexpression analyses suggest that an adjusted level of FRPR-19-dependent signaling in both FLP and *glr-1*-expressing interneurons is essential to maintain the responsiveness of the FLP neural pathway in order to support aversive behavior.

### FLP-8 and FLP-14 activate FRPR-19 *in vitro*

**Fig 4:**
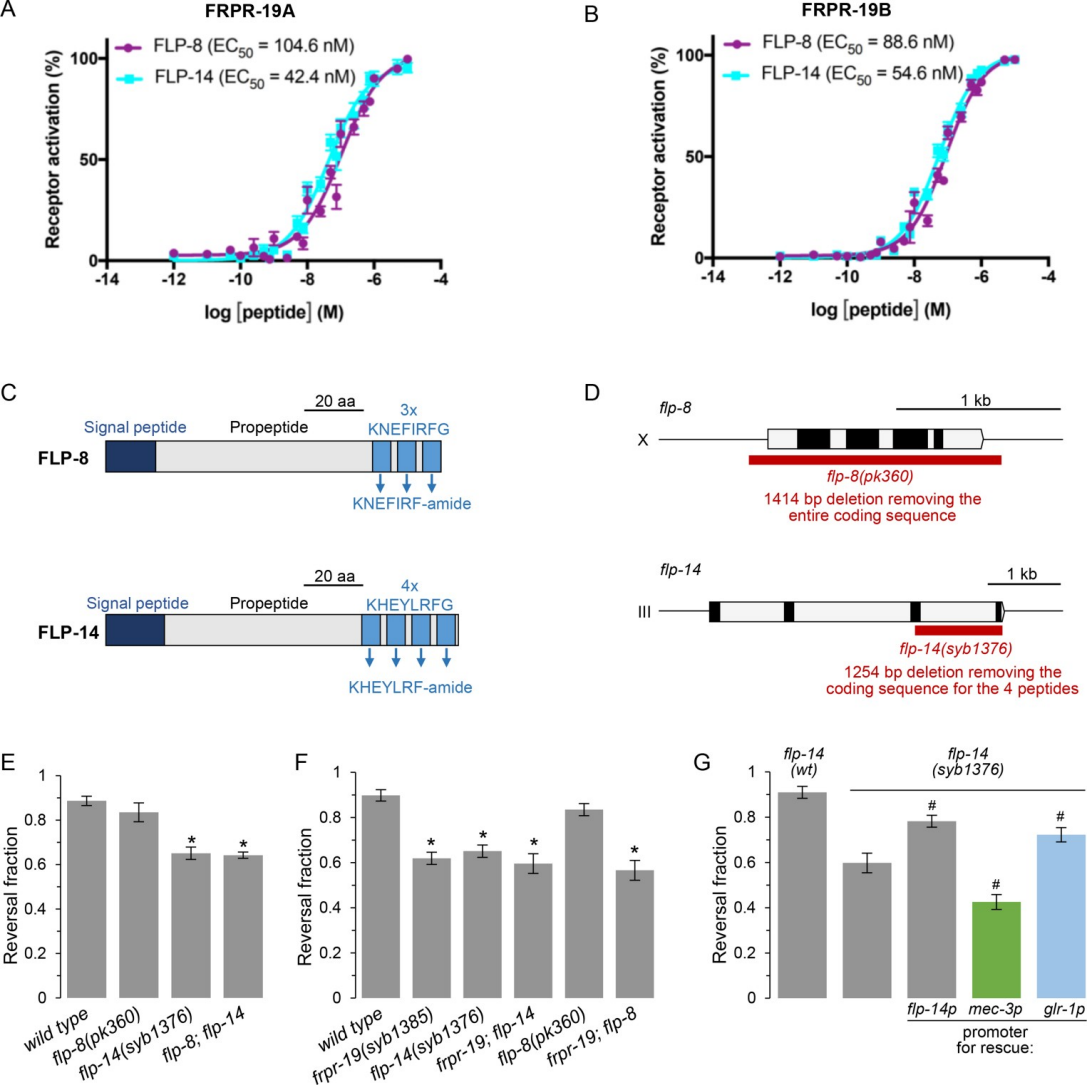
FLP-14 is an FRPR-19 ligand acting from interneurons to promote reversal. (A, B) Concentration-response curves of the mean calcium responses (% activation ± SEM) in CHO cells expressing either FRPR-19A (A) or FRPR-19B (B) for different concentrations of synthetic peptides FLP-8 (solid purple circles) or FLP-14 (solid cyan squares). Solid lines indicate curve fits to the data (*n* = 8). 95% confidence intervals (nM), FRPR-19A: FLP-8, 75.8–151.6; FLP-14, 32.4–56.7 and FRPR-19B: FLP-8, 71.9–110.9; FLP-14, 46.9–63.8. (C) Schematic of the FLP-8 and FLP-14 propeptide structure. (D) Schematic of the *flp-8* and *flp-14* gene topology. Open boxes: introns and UTRs. Closed boxes: coding exons. Red boxes: deletions in the indicated mutant alleles. (E, F, G) Fraction of FLP optogenetic stimuli producing a reversal response scored as in Fig 1 for the indicated genotypes. Epistasis analyses between *flp-8* and *flp-14* null mutations (E), between *flp-8*, *flp-14* and *frpr-19* null mutations (F), and cell-specific rescue in *flp-14* null mutants suggesting that *glr-1*-expressing interneurons represent a functionally relevant source of FLP-14 peptides (G). *, *p*<,01 versus wild type and #, *p* < .01 versus *flp-14* by Bonferroni contrasts.

FRPR-19 is an orphan receptor that is part of the FMRFamide-Like Peptide Receptor family [58]. In order to identify its ligands, we used a previously described calcium mobilization assay [59], in which the activation of candidate GPCRs heterologously expressed in mammalian cells can be monitored and libraries of chemically-synthesized neuropeptides screened. We tested a total of 344 peptides corresponding to detected or predicted products of *nlp* and *flp* neuropeptide-encoding genes, and separately assessed their ability to activate FRPR-19A and FRPR-19B. We identified FLP-8 and FLP-14 as two ligands of FRPR-19A and FRPR-19B with similar potency (Fig 4A and 4B). Neither peptide elicited a calcium response in cells transfected with an empty control vector. FLP-8 and FLP-14 are two well-conserved FMRFamide-like peptides with homologs in other nematodes, in which they were previously named AF1 and AF2, respectively. FMRFamide-like peptides are conventionally designated with the FLP acronym; not to be confounded with FLP neurons. FLP-8 and FLP-14 peptides are generated via the processing of propeptides, which contain three or four repetitions, respectively, of the peptide sequence in their C-terminal region (Fig 4C).

### A FLP-14/FRPR-19 signaling pathway regulates FLP neuron-dependent aversive behavior *in vivo*

Having shown that FLP-8 and FLP-14 are ligands for FRPR-19 *in vitro*, we next tested whether these neuropeptides take part in the FRPR-19-dependent aversive response control *in vivo*. To that end, we measured FLP-evoked reversals in *flp-8* and *flp-14* null mutants. We used a previously described *flp-8(pk360)* allele [60], in which the whole coding sequence is deleted, as well as a novel CRISPR/Cas9-generated *flp-14(syb1376)* allele, in which we deleted the last two *flp-14* exons containing the coding sequence for all four peptide repetitions (Fig 4D). Of the two mutations, only *flp-14* mutation caused a decreased reversal response (Fig 4E). The defect in a *flp-8;flp-14* double mutant was identical to that in *flp-14* single mutant (Fig 4E). These results indicate that *flp-14*, but not *flp-8*, is essential to control FLP neuron-evoked aversive responses and are in line with a model in which FLP-14 could represent a functional FRPR-19 ligand *in vivo*, relevant to this phenotype. To further substantiate this model, we assessed potential genetic interactions between *frpr-19* and each of the two neuropeptide genes. We could not observe a further reversal response decrease in double *frpr-19;flp-14* mutants, whose response was indistinguishable from that in the single mutants (Fig 4F). These results indicate that *frpr-19* and *flp-14* are part of the same genetic pathway. In contrast, the *frpr-19*;*flp-8* double mutants had the same phenotype as the *frpr-19* mutants, but not as the *flp-8* single mutants, suggesting that *frpr-19* and *flp-8* do not function in the same pathway (Fig 4F). Collectively, these results suggests that a FLP-14/FRPR-19 signaling pathway modulates the responsiveness of the neural pathway downstream of FLP nociceptor activation.

### *flp-14* expression in interneurons promotes FLP neuron-dependent aversive behavior

Previous reporter gene and scRNA-seq analyses have shown that *flp-14* is expressed in several neurons, with the most robust expression in RMG, RID, IL2, AVD and AVM neurons [56,61]. scRNA-seq data suggest little or no expression in FLP, with an expression value almost 300 times lower than that in the top expressing neuron (RMG) [56]. Consistent with an absence of *flp-14* expression in FLP, we found no evidence for a *[flp-14p*::*flp-14*::*SL2*::*mCherry]* reporter expression in these neurons. This same construct could significantly rescue the defect in *flp-14(syb1376)* mutants, suggesting that *flp-14* is likely to act in other neurons in order to regulate FLP neuron-dependent aversive response. To identify the relevant locus of action of the *flp-14* gene, we performed additional rescue experiments with cell-specific promoters. A significant rescue effect was produced with the *glr-1* promoter (Fig 4G). This promoter is expressed in all the interneurons downstream of FLP in the reversal-promoting pathway, including in AVD where *flp-14* is strongly expressed in wild type. In contrast, using the *mec-3* promoter to drive the *flp-14* expression in FLP did not rescue the *flp-14* mutant defect, but instead caused a further decrease in reversal response. This phenotypic aggravation might result from the aberrant FLP-14 expression in neurons where it is not normally present, at least at a high expression level.

Taken together, our results suggest a model in which FLP-14 released from *glr-1-*expressing interneurons promotes the responsiveness of the FLP neuron-dependent reversal pathway, and may involve a feedback on FRPR-19-expressing cells including in FLP primary sensory neurons.

### FLP-14/FRPR-19 signaling prevents the habituation to repeated noxious heat and harsh touch stimulations

The above-reported optogenetic experiments involving precisely targeted stimulatory inputs are ideal for a quantitative functional dissection of the neural pathway linking FLP activation with reversal behavior and to highlight specific modulatory mechanisms. Nevertheless, selective optogenetic stimulation remains an artificial situation. We therefore wanted to assess the role of FRPR-19 signaling in modulating the aversive response caused by natural noxious stimuli sensed by FLP neurons, namely high temperature and harsh mechanical stimuli [38,42,47].

**Fig 5:**
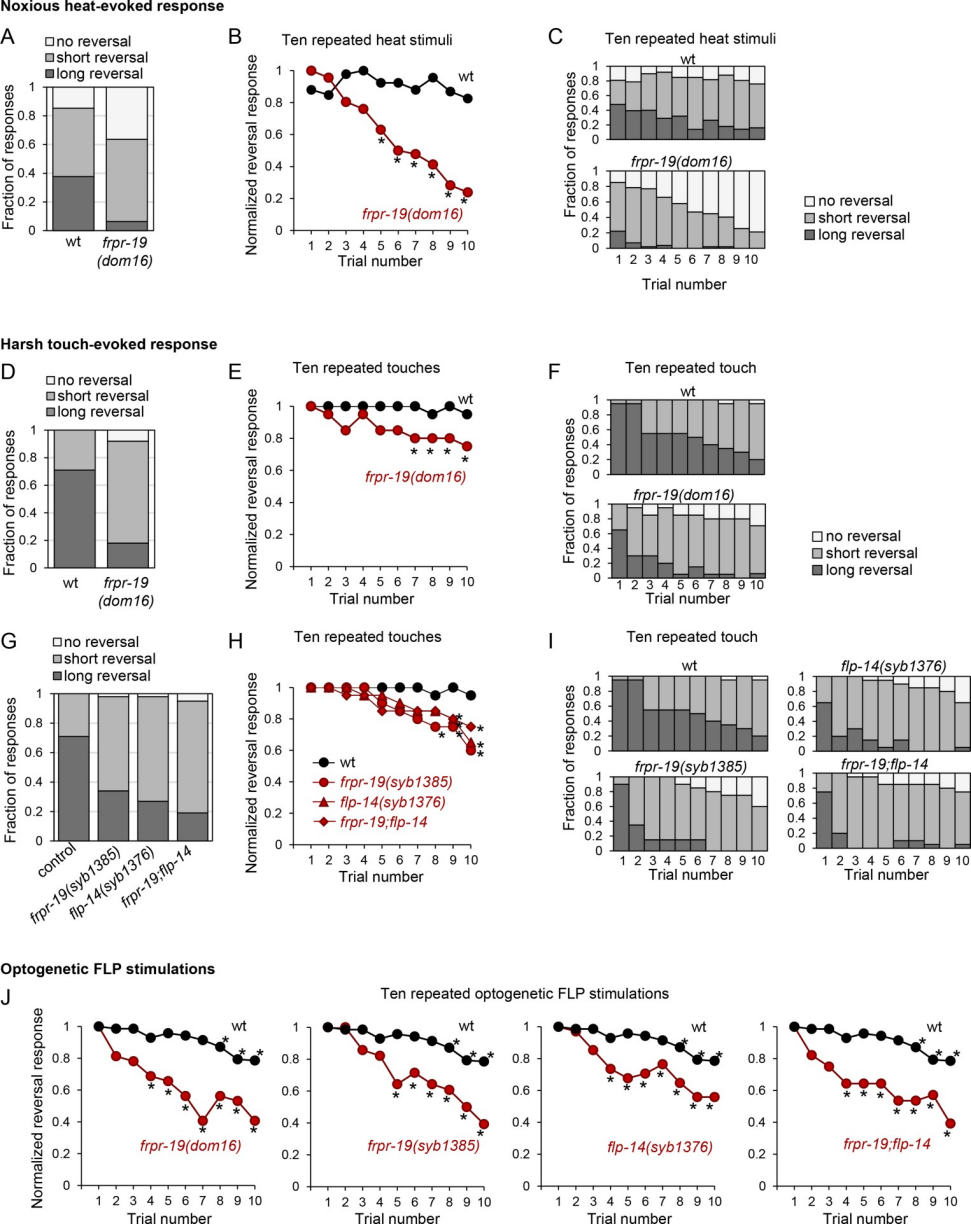
Mutations affecting the FLP-14/FRPR-19 pathway cause fast habituation of responses to noxious heat and harsh touch. (A, B, C) Impact of *frpr-19* mutation on noxious heat-evoked reversals. Reversal length comparison, based on a five-trial assay (A) or across a longer habituation protocol of ten repeated stimuli (C). Normalized reversal response, expressed as fraction of the maximal response rate and showing faster habituation in *frpr-19* mutant (B). (D-I). Impact of *frpr-19* and *flp-14* mutations on harsh touch-evoked reversals. Reversal length comparison, based on a five-trial assay (D, G) or across a longer habituation protocol of ten repeated stimuli (F, I). Normalized reversal response, expressed as fraction of the maximal response rate and showing faster habituation in *frpr-19* mutant (E, H). (J) Impact of *frpr-19* and *flp-14* mutations on optogenetic FLP stimulation-evoked reversals in a ten-stimuli habituation protocol, showing a faster habituation in the mutants. *, *p* < .01 versus corresponding wild type data point by Bonferroni contrasts (B, E, H, J). The data without normalization corresponding to panels B, E, H and J are presented in S3 Fig.

We first compared noxious heat-evoked reversals in wild type and *frpr-19(dom16)* mutants. In an initial measure set, animals were stimulated five times in a row with 4 s stimuli (each causing a 1.4°C/s temperature increase) with an inter-stimulus interval of 20 s. This stimulation protocol was chosen because, in wild type, it produces a robust, yet non-saturating response (~85% overall responsiveness), with no obvious signs of habituation. On a qualitative standpoint, the response includes both short (≤2 head swings) and long reversals (>2 head swings) in similar proportions (Fig 5A). We observed a significantly decreased response in *frpr-19* mutants, who responded to only 62% of the trials and produced shorter reversals (Fig 5A). We also noted that the defect was getting stronger over the 5 trials, which prompted us to more extensively examine the response habituation with 10 stimuli series. In wild type, repeated stimulations did not decrease the reversal response frequency, but caused a slight, progressive decrease in the fraction of long reversals (Fig 5B and 5C). In contrast, *frpr-19* mutants almost never produced long reversals after the third stimulus and their response rate was rapidly declining to reach values <30% after 10 stimuli (Figs 5B and 5C and S3A). This very fast habituation phenotype in *frpr-19* mutants suggests that intact FRPR-19 signaling is needed to prevent an habituation effect to repeated noxious heat stimuli and maintain avoidance behaviors.

Next, in order to assess FRPR-19 signaling contribution to the regulation of mechanical stimulus avoidance, we conducted similar analyses with harsh touch stimuli delivered on the animal head. Wild type animals produced reversal responses to almost every stimulus (Figs 5D and 5E and S3B). While repeated simulations did not affect the fraction of reversal responses, we observed a marked shortening of the reversals after a ten stimuli series (Figs 5E and S3B). The *frpr-19(dom16)* mutation caused a significant response reduction, which included an overall shortening of reversals (Fig 5D) and faster habituation to repeated stimuli (with both decreasing response rate and faster reversal shortening, Fig 5D and 5E). To further test the implication of the FLP-14/FRPR-19 pathway in regulating harsh touch-evoked response, we performed similar analyses with *frpr-19(syb1385)* and *flp-14(syb1376)* null mutants, as well as double mutants. The harsh touch response phenotypes in these mutants (Figs 5G–5I and S3B) were strikingly similar to that caused by the *frpr-19(dom16)* mutation (Figs 5C–5F and S3B). In addition, there was no additive effect between mutations in the two genes. This is in line with a model in which FLP-14 and FRPR-19 work in the same pathway in order to promote reversal response.

Finally, we investigated whether a defect in the FLP-14/FRPR-19 pathway would also accelerate the habituation to repeated optogenetic stimulation of FLP. In addition to a decreased responsiveness in naïve animals (S3C Fig, first stimulus), the mutations in *flp-14* and *frpr-19* alone or in combination caused an accelerated habituation phenotype (Fig 5J), like for harsh touch-evoked reversal (Fig 5E and 5H). Because optogenetic stimulations bypass sensory transduction, these results suggest that the modulatory action of the FLP-14/FRPR-19 pathway takes place downstream of stimulus-evoked FLP neuron depolarization.

## Discussion

Habituation processes taking place in sensory systems in response to persistent or repeated stimuli constitute a mean to ignore irrelevant stimuli. We all have experiences of sensory plasticity in response to innocuous stimuli, for example when going for a swim and acclimating to cool water or when our perception of a persistent smell rapidly fades away. Habituation to noxious stimuli is less obvious in daily life, presumably because we tend to avoid repeated or persistent exposure. Nociceptive habituation phenomena are best evidenced in human by using relatively long-lasting experimental protocols occurring over hours or days [62]. In contrast, over a shorter timeframe, repeated stimulations do not typically cause strong habituation, and sometime rather elicit sensitization [62–65]. Comparatively slow habituation kinetics have been documented for nociceptive response in a variety of organisms from human to *C*. *elegans*, and seem to be part of a generally valid ecological strategy [46,48,66–69]. Indeed, animals should not rapidly ignore signals indicative of a potential damage, but do so only when the cost of withdrawal responses themselves becomes too high (e.g., when preventing animal feeding or long-range exploration). The underlying processes causing a lower habituation propensity in nociceptive pathways are poorly understood. Do slow-habituating nociceptive pathways lack pro-habituation mechanisms, or instead, do they engage active anti-habituation mechanisms? Here, we identify a neuropeptide-based signaling pathway that is essential to prevent fast habituation in the FLP nociceptive pathway of *C*. *elegans*. Our findings provide an example where the robust response and low habituation propensity commonly seen in nociceptive pathways are not passive, intrinsic properties, but actually rely on an active cell-to-cell communication via neuropeptidergic signaling between interneurons and primary sensory neurons.

We found that mutations in the *frpr-19* and *flp-14* genes impair reversal behavior in response to the selective optogenetic activation of FLP, as well as in response to noxious heat and harsh touch stimuli. The mutant phenotypes were similar across the different stimulation modalities and comprised (i) a shortening of reversal responses in naïve mutant animals and (ii) a faster habituation. However, the magnitude of these effects differed according to the specific stimulation protocols. These quantitative differences may be due to the different activation levels produced by individual stimuli of different modalities, to the different interstimulus intervals (which varied between assay types), and/or to the different neural circuits engaged by each stimulation modality. For example, the milder habituation enhancement seen for harsh touch, as compared to optogenetic FLP activation and noxious heat stimuli, may relate to the fact that harsh touch stimuli can activate a distinct, larger set of sensory neurons to trigger reversal than the other stimulus types. In future studies, a more systematic analysis will be needed to address the impact of stimulus intensities, of interstimulus intervals and dissect the contribution of additional sensory neurons working in parallel to FLP.

**Fig 6:**
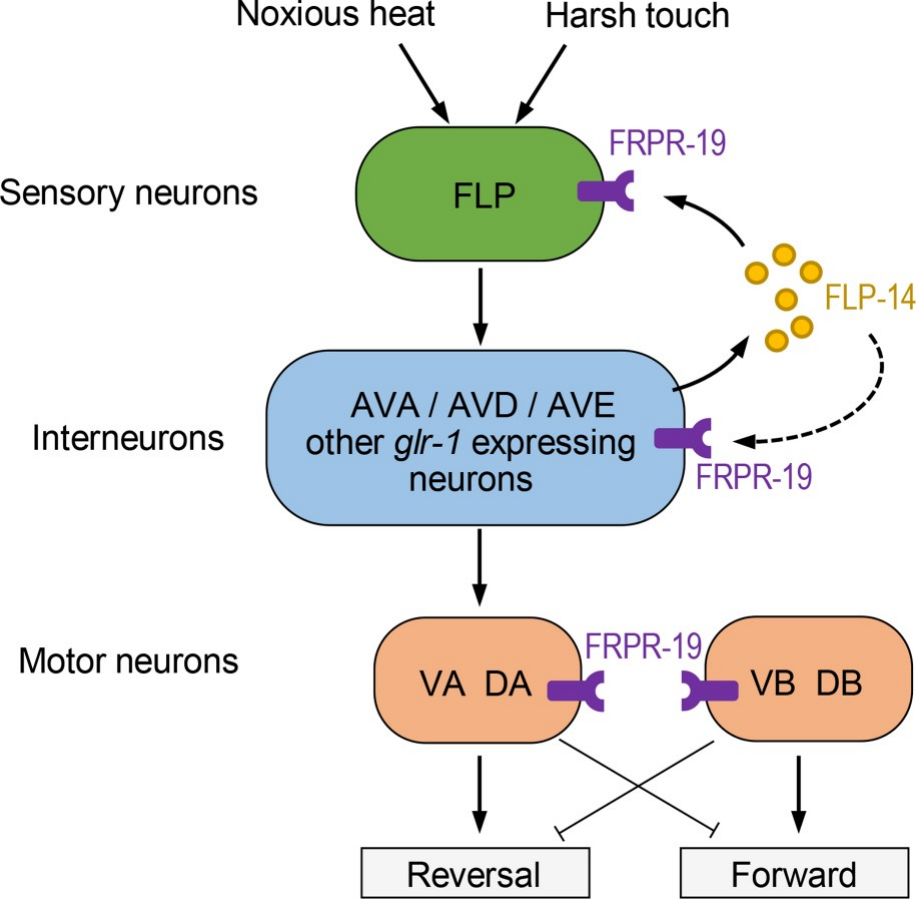
Proposed model for the FLP-14/FRPR-19 signaling modulatory action in the circuit controlling reversal behavior. Schematic of the reversal circuit downstream of FLP sensory neurons showing the expression of FRPR-19 and the potential source of FLP-14 peptides. A positive feedback via an excitatory FLP-14/FRPR-19 peptide signaling could contribute to the maintenance of a high responsiveness under a regime of repeated noxious stimuli. Presumably, a stimulus-dependent activation of the FLP-14/FRPR-19 positive feedback loop may counteract antagonistic habituation mechanisms. The place of action of these pro-habituation mechanisms is unknown. Additional FLP-14 sources are not ruled out by our data (not depicted).

Based on expression and cell-specific rescue data, we propose a circuit model through which the FLP-14/FRPR-19 signaling could regulate the activity of the neural pathway linking FLP to reversal behaviors (Fig 6). While they do not exclude other potential FLP-14-emitting cells, our data show that *glr-1*-expressing interneurons constitute a functionally relevant source of FLP-14 neuropeptides. These interneurons include all the postsynaptic partners of FLP and in particular AVD where high levels of *flp-14* mRNA were previously detected [56]. FLP-14 can then activate FRPR-19, which is relatively broadly expressed in the reversal pathway, including in FLP sensory neurons, in several *glr-1*-expressing interneurons, as well as in motor neurons controlling both forward (VB/DB) and backward locomotion (VA/DA). The activity of FRPR-19 in FLP and downstream interneurons is probably relevant for the activity-dependent regulation of reversal responses, as indicated by a partial rescue of the mutant phenotype when restoring FRPR-19 expression in either of these cell types. Therefore, we propose that FLP-14 signals function both as a feedback mechanism on FLP neurons and within its downstream interneurons (Fig 6). There is no documented chemical synapse between *glr-1*-expressing interneurons and FLP, which could be engaged in a synaptic feedback to FLP [70]. However, the relatively high potency of FLP-14 for activating FRPR-19 is compatible with an extra-synaptic action of FLP-14.

FLP-14 is phylogenetically related to the RFamide related peptides, which are broadly conserved across the animal kingdom [58,71], and which were shown to act as neuromodulators in the pain pathway in mammals [72]. In *C*. *elegans*, previous studies indicated that FLP-14 could regulate methyl salicylate avoidance [73], and act on B and D motor neurons to promote forward locomotion and help sustain long-lasting behavioral states [61]. Furthermore, exogenous peptide application studies showed that FLP-14 is an excitatory peptide in the pharyngeal system of *C*. *elegans* [74]. Likewise, the excitatory effect of FLP-14/AF2 homologous neuropeptide was shown at the neuromuscular junction of different nematode species, where it potentiates transmission via a presynaptic effect [75,76]. Because our data show that the loss of FLP-14 reduces FLP-evoked reversal and that this peptide acts in cells that positively regulate the reversal pathway, we conclude that FLP-14 is most likely excitatory and that its receptor FRPR-19 should up-regulate neural activity. Our data show that the FLP-14/FRPR-19 signaling pathway is required to maintain the response to optogenetic activation of FLP neurons, which bypasses sensory transduction. Therefore FLP-14/FRPR-19 signaling most likely acts by potentiating neurotransmission rather than modulating sensory transduction. Further studies will be needed to determine the mechanism through which this pathway modulates neurotransmission, notably whether it produces a quantitative (transmission volume) or a qualitative (type of transmitter) impact.

Whereas FLP-14 was previously shown to be a weak, partial agonist of the NPR-1 receptor *in vitro*, evidence for the relevance of this potential interaction *in vivo* is lacking [73]. Here, we identify FRPR-19 as a physiologically relevant receptor for FLP-14, with a relatively high potency and half maximal effective concentrations (EC_50_) in the nanomolar range, typical for neuropeptide receptors [77]. We are not aware of any previous studies having addressed the function of FRPR-19 receptors. The *frpr-19(dom16)* allele recovered in our genetic screen only affects the FRPR-19B isoform, and removes two conserved residues at its C-terminus, which are critical for its function. Because this allele produced the same impact as a predicted null allele or as an engineered deletion causing the selective loss of *frpr-19b*, we favor a model in which this allele represents a loss-of-function. The slight semi-dominance of the *dom16* allele and the non-redundant function of FRPR-19A and B isoforms could reflect the requirement for a tightly adjusted expression/activity FRPR-19 level, as well as for a well-balanced expression between the two isoforms. One possible explanation could be that FRPR-19A and B isoforms would function as heterodimers *in vivo*. Heterodimerization could be essential for proper signaling activity or for appropriate trafficking to and/or from the cell surface, like previously reported for other GPCRs [see 78 for a review].

The mechanism through which overexpression of FRPR-19A or B isoforms (alone or in combination) impairs behavior is not known at this stage. These impairments may reflect an aberrantly high or persisting FRPR-19 signaling. If one assumes an excitatory role of this pathway (as discussed above), then it is conceivable that a stimulus-independent chronic activation of this positive feedback might cause a paradoxical downregulation of the pathway responsiveness by triggering long-term habituation mechanisms downstream in the circuit. Alternatively, we cannot rule out that FRPR-19 overexpression might interfere with the expression or function of additional receptors. Regardless of the underlying mechanism, it is remarkable that both up-regulation and down-regulation of FRPR-19 receptor function can reduce the responsiveness in the FLP pathway. FRPR-19 might therefore serve a dual role, producing both pro-nociceptive or anti-nociceptive actions in different context and/or over different timescales. At the same time, it represents a fragile regulatory node in the nociceptive pathway. If similar nodes exist in mammals, they would constitute promising targets to trigger anti-nociceptive effects with either agonists or antagonists.

In conclusion, we have identified an excitatory FLP-14/FRPR-19 pathway functioning to maintain high nociceptive responsiveness in *C*. *elegans*. Our results highlight the importance of active communication mechanisms counteracting habituation in nociceptive pathways and set a potent *in vivo* model to decipher the contribution of neuropeptide signaling in nociceptive plasticity.

## Methods

### *C*. *elegans* strains and growth conditions

The *C*. *elegans* strains used in this study are listed in S1 Table. All CRISPR-generated strains (Suny Biotech, Fuzhou, China) were outcrossed four times with wild type prior to analysis. Worms were grown on nematode growth media (NGM) plates with OP50 *Escherichia coli* as previously described [79]. For optogenetic experiments, we prepared plates with or without ATR. When ATR was included, we added 0.1% (v/v) of ATR stock (100 mM, in ethanol) to the OP50 bacteria suspension, and seeded each NGM agar plate (60 mm diameter and 15 mm height, containing 8 ml NGM) with 300 μl of this mix.

### Behavioral assays

#### Worm preparation and reversal scoring

All experiments were performed blindly with respect to the genotypes. All experimental replicates were obtained over at least 3 independent days and adult worms were used either synchronized by bleaching or picked as L4 larvae on NGM plates one day before the experiments. Stimulations (light, touch or heat) were delivered as detailed below, scoring was done manually and any backward movement taking place during the stimulation (or immediately after, for harsh touch) was counted as a positive response (reversal). For most optogenetic experiments, animals were stimulated five times in a row. Each animal received a response score over these five trials. Scores were then averaged over the animals for each genotype. For habituation experiments, animals were stimulated ten times in a row and response scores were recorded on a trial-by-trial basis. In order to focus on the habituation kinetics, reversal fraction was normalized as fraction of the maximal response (*Normalized reversal response* in (Fig 5B, 5E, 5H and 5J). Data without normalization are also presented (S3 Fig). For reversal length categorization, the number of body bends during reversals were counted to classify reversals as long (more than 2 body bends) or short (2 body bends or less).

#### Light stimulation for optogenetic FLP stimulation

FLP optogenetic activation was performed as previously described [47]. Single forward moving animals were illuminated with 61 W/m^2^ blue light during 0.5 s. Between two stimuli, we waited for the worm to restart forward locomotion. The interstimulus interval was not fixed but in a range between 5 and 10 s.

#### Heat stimulation

Noxious heat stimuli were delivered as previously described [47]. 50–150 animals were stimulated with four 100W infra-red lamps heating the plate at a rate of ~1.4°C/s during 4 seconds. Videos were recorded with a DMK23UP1300 camera (The Imaging Source, Germany) and we manually scored the heat-evoked responses over 10 trials with an interstimulus interval of 20 s.

#### Harsh touch stimulation

Harsh touch was delivered with a platinum wire pick as previously described [42,47]. The stimulus was applied from above the animals by pressing down with the edge of the pick with an estimated force of 100 μN on the head of a forward moving adult animal. Between two stimuli, we waited for the worm to restart forward locomotion. The interstimulus interval was not fixed but in a range between 5 and 10 s.

### Genetic screen and mutation mapping

The genetic screen and mutation mapping was performed as previously described [47]. In brief, the progeny (F2 generation) of EMS-mutagenized DAG356 animals were screened for impaired light-evoked reversal, mutant lines isolated and the phenotype confirmed in subsequent generations. The *dom16* mutation was mapped after five rounds of iterative backcross with DAG356, via EMS-density mapping.

### Microscopy

For fluorescent reporter imaging, we used either a Zeiss Axio Plan 2 fluorescence microscope (40x air objective, NA = 0.95) or a Leica TCS SPE-II confocal microscope (APO 40x oil objective, NA1.15), equipped with a 488 nm wavelength diode laser and an ET525/50m emission filter. Z-stack images were acquired across whole animal thickness and maximal intensity projections are depicted.

### Screen for FRPR-19A and FRPR-19B ligands

The GPCR activation assay was performed as previously described [59]. Briefly, CHO-K1 cells stably expressing the luminescent Ca^2+^ indicator aequorin and the promiscuous G_α16_ protein (ES-000-A24 cell line, PerkinElmer) were transiently transfected with *frpr-19a*/pcDNA3.1 or *frpr-19b*/pcDNA3.1. Cells were transfected with Lipofectamine LTX and Plus reagent (Invitrogen) and grown overnight at 37°C. After 24 hours, they were shifted to 28°C overnight. On the day of the assay, transfected cells were collected in bovine serum albumin (BSA) medium (DMEM/F12 without phenol red with L-glutamine and 15 mM HEPES, Gibco, supplemented with 0.1% BSA) at a density of 5 million cells per mL, and loaded with 5 μM coelenterazine h (Invitrogen) for 4 hours at room temperature. Compound plates containing synthetic peptides in DMEM/BSA were placed in a MicroBeta LumiJet luminometer (PerkinElmer). After loading, the transfected cells were added at a density of 25,000 cells/well, and luminescence was measured for 30 s at a wavelength of 469 nm. After 30 s, 0.1% triton X-100 (Merck) was added to lyse the cells, resulting in a maximal Ca^2+^ response that was measured for 30 s. Concentration-response measurements were done in triplicate on at least two independent days. For each peptide concentration, a relative calcium response (%) compared to the maximum peptide-evoked response (100% activation) was calculated. Concentration-response data were then fitted in function of log[peptide]. EC_50_ values were calculated from concentration-response curves in GraphPad Prism 7 by fitting a 4-parameter concentration-response curve.

### Transgene construction and transgenesis

DNA prepared with a GenElute HP Plasmid miniprep kit (Sigma) was microinjected in the gonad to generate transgenic lines according to a standard protocol [80]. We used a [*unc- 122p*::*GFP*] (gift from Piali Sengupta; Addgene plasmid # 8937 [81]) co-injection marker to identify transgenic animals. The concentration of each plasmid in the injection mixes was 20 ng/μl for the co-marker and for expression constructs. The only exception was for some *frpr-19p*-containing lines, for which we also injected a lower concentration (2 ng/μl, as specified in S1 Table).

#### Fosmid

WRM0623dC10 (*frpr-19*-containing fosmid) was obtained from Source BioScience (Nottingham, UK).

#### Promoter plasmids (MultiSiteGateway slot 1)

Entry plasmids containing specific promoters were constructed by PCR from N2 genomic DNA with primers flanked with attB4 and attB1r recombination sites and cloned into pDONR-P4-P1R vector (Invitrogen) by BP recombination. Primer sequences were the following:

dg721 [slot1 Entry frpr-19p]
attB4frpr-19_F: ggggacaactttgtatagaaaagttgatgtgtttgtcaataggaaaccagtttc
attB1rfrpr-19_R: ggggactgcttttttgtacaaacttgttcttgcggcggtgatgggta
dg858 [slot1 Entry flp-14p]
attB4flp-14_F: ggggacaactttgtatagaaaagttgatcgattttccgttcctggtctc
attB1rflp-14_R: ggggactgcttttttgtacaaacttgtgaagagagctctcagttggag

The generation of dg68 [slot1 Entry mec-3p] was previously described [82]. The *glr-1p* entry clone, dg25 [slot1 Entry glr-1p], was a gift from Kaveh Ashrafi [57].

#### Coding sequence plasmids (MultiSiteGateway slot 2)

Entry plasmids containing specific coding DNA sequences (cds) or genomic sequence (gs) were constructed by PCR from N2 cDNA or N2 genomic DNA with primers flanked with attB1 and attB2 recombination sites and cloned into pDONR_221 vector (Invitrogen) by BP recombination. Primer sequences were the following:

dg852 [slot2 Entry frpr-19gs]
attB1frpr-19AB_F: ggggacaagtttgtacaaaaaagcaggcttaatgacgacggacgctaatttc
attB2frpr-19B_R: ggggaccactttgtacaagaaagctgggtttcaagtttgactcttctcgagac
dg722 [slot2 Entry frpr-19Acds]
attB1frpr-19AB_F: ggggacaagtttgtacaaaaaagcaggcttaatgacgacggacgctaatttc
attB2frpr-19A_R: ggggaccactttgtacaagaaagctgggttctaattgagtacggaactaacgag
dg723 [slot2 Entry frpr-19Bcds]
attB1frpr-19AB_F: ggggacaagtttgtacaaaaaagcaggcttaatgacgacggacgctaatttc
attB2frpr-19B_R: ggggaccactttgtacaagaaagctgggtttcaagtttgactcttctcgagac
dg859 [slot2 Entry flp-14cds]
attB1flp-14_F: ggggacaagtttgtacaaaaaagcaggcttaatatgatgatctgcctgccca
attB2flp-14_R: ggggaccactttgtacaagaaagctgggttaaccaacgacaagatttattacaatttc

The generation of dg398 [slot2 Entry mNeongreen3xFLAG] was previously described [83].

#### 3’ UTR and tagging plasmids (Multi-site Gateway slot3)

mg277 [SL2::mCherry] was previously described [82].

mg211 [EntrySlot3unc-54UTR] (aka pMH473) was a gift from Marc Hammarlund.

#### Expression plasmids used for transgenesis

dg401 [mec-3p::mNeongreen3xFLAG::unc-54UTR] was previously created through a LR recombination reaction between dg68, dg398, mg211, and pDEST-R4-P3.

dg732 [frpr-19p::frpr-19Acds::SL2::mCherry] was created through a LR recombination reaction between dg721, dg722, mg277, and pDEST-R4-P3.

dg733 [frpr-19p::frpr-19Bcds::SL2::mCherry] was created through a LR recombination reaction between dg721, dg723, mg277, and pDEST-R4-P3.

dg853 [glr-1p::frpr-19gs::SL2::mCherry] was created through a LR recombination reaction between dg25, dg852, mg277, and pDEST-R4-P3.

dg854 [mec-3p::frpr-19gs::SL2::mCherry] was created through a LR recombination reaction between dg68, dg852, mg277, and pDEST-R4-P3.

dg855 [frpr-19p::frpr-19gs::SL2::mCherry] was created through a LR recombination reaction between dg721, dg852, mg277, and pDEST-R4-P3.

dg860 [flp-14p::flp-14cds::SL2::mCherry] was created through a LR recombination reaction between dg859, dg, mg277, and pDEST-R4-P3.

dg884 [glr-1p::flp-14cds::SL2::mCherry] was created through a LR recombination reaction between dg25, dg859, mg277, and pDEST-R4-P3.

dg885 [mec-3p::flp-14cds::SL2::mCherry] was created through a LR recombination reaction between dg68, dg859, mg277, and pDEST-R4-P3.

### Transcript sequencing

Transcript sequencing in CRISPR/Cas9-edited lines was used to verify the presence/absence of *frpr-19* isoforms and their splicing as described in Fig 2A. cDNA libraries were prepared as previously described from mixed stage worm populations [84], PCR-amplified using attB1frpr-19AB_F/attB2frpr-19A_R and attB1frpr-19AB_F/attB2frpr-19B_R primer pairs, respectively, and sequenced with frpr-19F6 primer (TGTAACACTCTTGCATTCGTTTGCAA).

## Supporting information

S1 Fig Alternative splicing in the *frpr-19* gene is conserved across *Caenorhabditis* species. (A) Genomic sequences and alternate exon definition a the 3’ end of the *frpr-19* gene coding region in the indicated *Caenorhabditis* species. Upper case: exonic sequence; lower case: intronic sequence; yellow: constitutive exon; blue: alternative exon sequence in isoform a; green: alternative exon sequence in isoform b. (B) Alignment of the predicted FRPR-19A/B amino acids sequence in the indicated *Caenorhabditis* species, highlighting the high conservation in each isoform. Color coding corresponding to respective exonic sequence in panel A.
(TIF)

S2 Fig FRPR-19 overexpression using the *frpr-19p* promoter decreases FLP neuron-dependent reversals in a dose-dependent manner in the *frpr-19* null background. Fraction of FLP optogenetic stimuli producing a reversal response, scored as in Fig 1, in the *frpr-19(syb1385)* null background mutants containing overexpression transgenes as multi-copy extrachromosomal arrays. *frpr-19gs* (genomic sequence); Micro-injected DNA concentrations for each plasmid are indicated. *, *p* < .01 versus non-transgenic *frpr-19* control and #, *p* < .01 between the two DNA concentrations by Bonferroni contrasts.
(TIF)

S3 Fig Reversal rate scored during repeated stimulation protocols without normalization. Fraction of stimuli producing a reversal response, scored as in Fig 5, but presented without normalizing to the maximal response. (A) Noxious heat stimuli. Data corresponding to Fig 5B. (B) Harsh touch stimuli. Data corresponding to Fig 5E and 5H. For harsh touch stimuli, the normalization had no impact because the maximal response was 100% for every genotype. Therefore, the harsh touch data series are identical in the two figures. (C) Optogenetic stimuli. Data corresponding to Fig 5J.
(TIF)

S1 Table Strain list. (DOCX)

S1 File Raw data and summary statistics for the data reported in the figures. (XLSX)
