# Supplementary figures and images for: Signaling via the FLP-14/FRPR-19 neuropeptide pathway sustains nociceptive response to repeated noxious stimuli in C. elegans
Source: PLoS Genet. 2021 Nov 8;17(11):e1009880. doi: 10.1371/journal.pgen.1009880 (PMC8601619; doi:10.1371/journal.pgen.1009880)

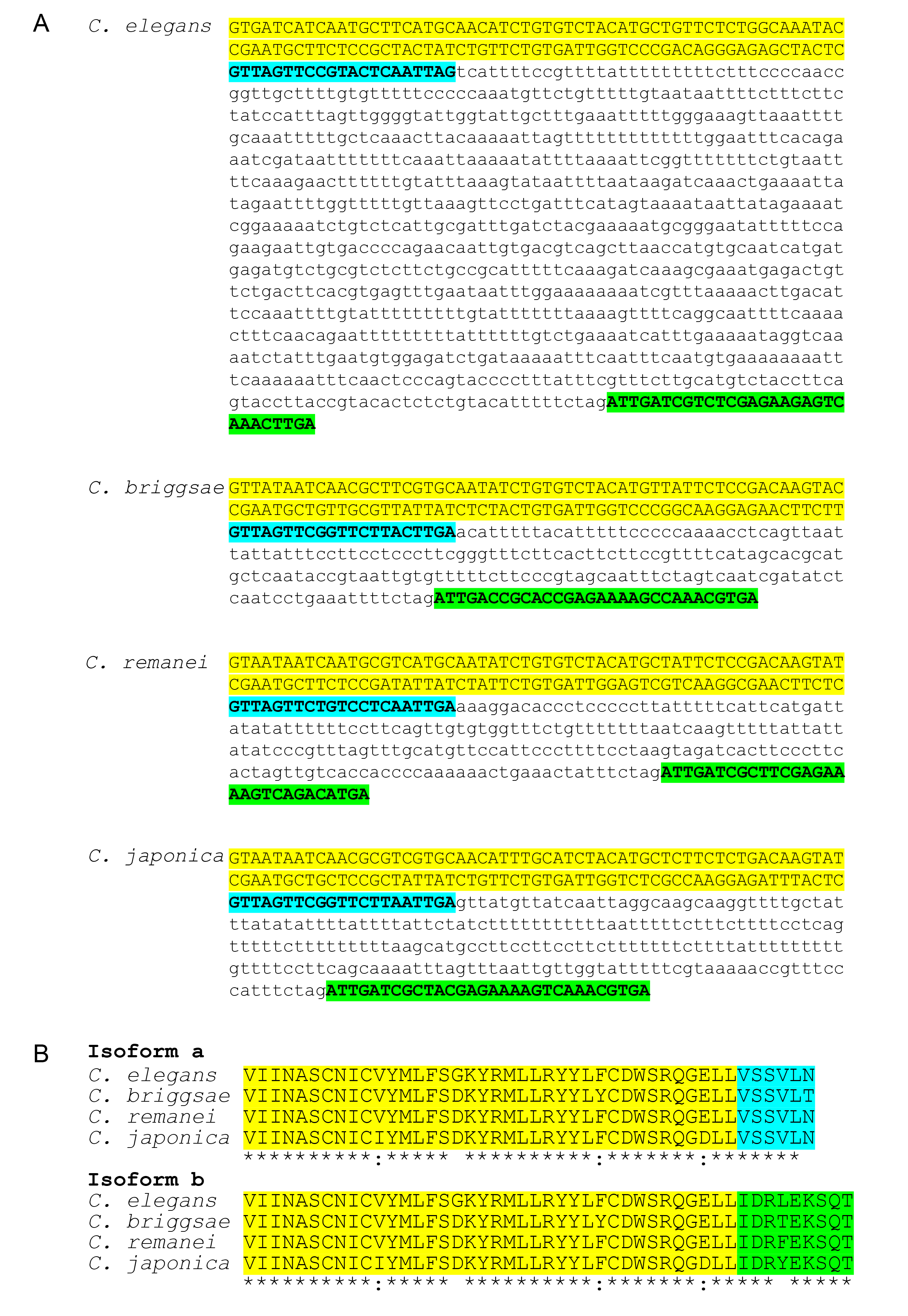

Supplement: S1 Fig — (A) Genomic sequences and alternate exon definition a the 3’ end of the frpr-19 gene coding region in the indicated Caenorhabditis species. Upper case: exonic sequence; lower case: intronic sequence; yellow: constitutive exon; blue: alternative exon sequence in isoform a; green: alternative exon sequence in isoform b. (B) Alignment of the predicted FRPR-19A/B amino acids sequence in the indicated Caenorhabditis species, highlighting the high conservation in each isoform. Color coding corresponding to respective exonic sequence in panel A. (TIF) [file pgen.1009880.s001.tif]

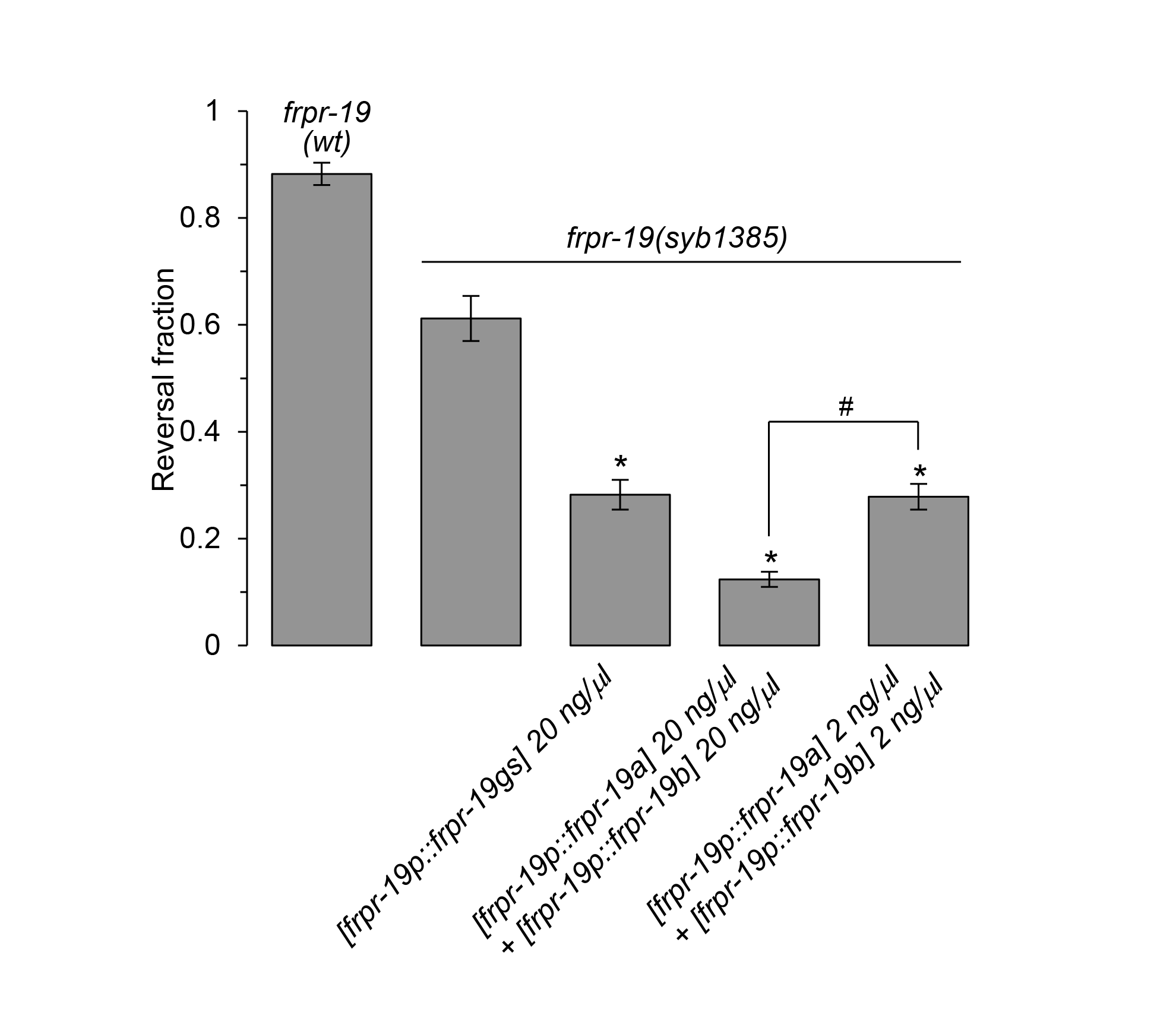

Supplement: S2 Fig — Fraction of FLP optogenetic stimuli producing a reversal response, scored as in Fig 1, in the frpr-19(syb1385) null background mutants containing overexpression transgenes as multi-copy extrachromosomal arrays. frpr-19gs (genomic sequence); Micro-injected DNA concentrations for each plasmid are indicated. *, p < .01 versus non-transgenic frpr-19 control and #, p < .01 between the two DNA concentrations by Bonferroni contrasts. (TIF) [file pgen.1009880.s002.tif]

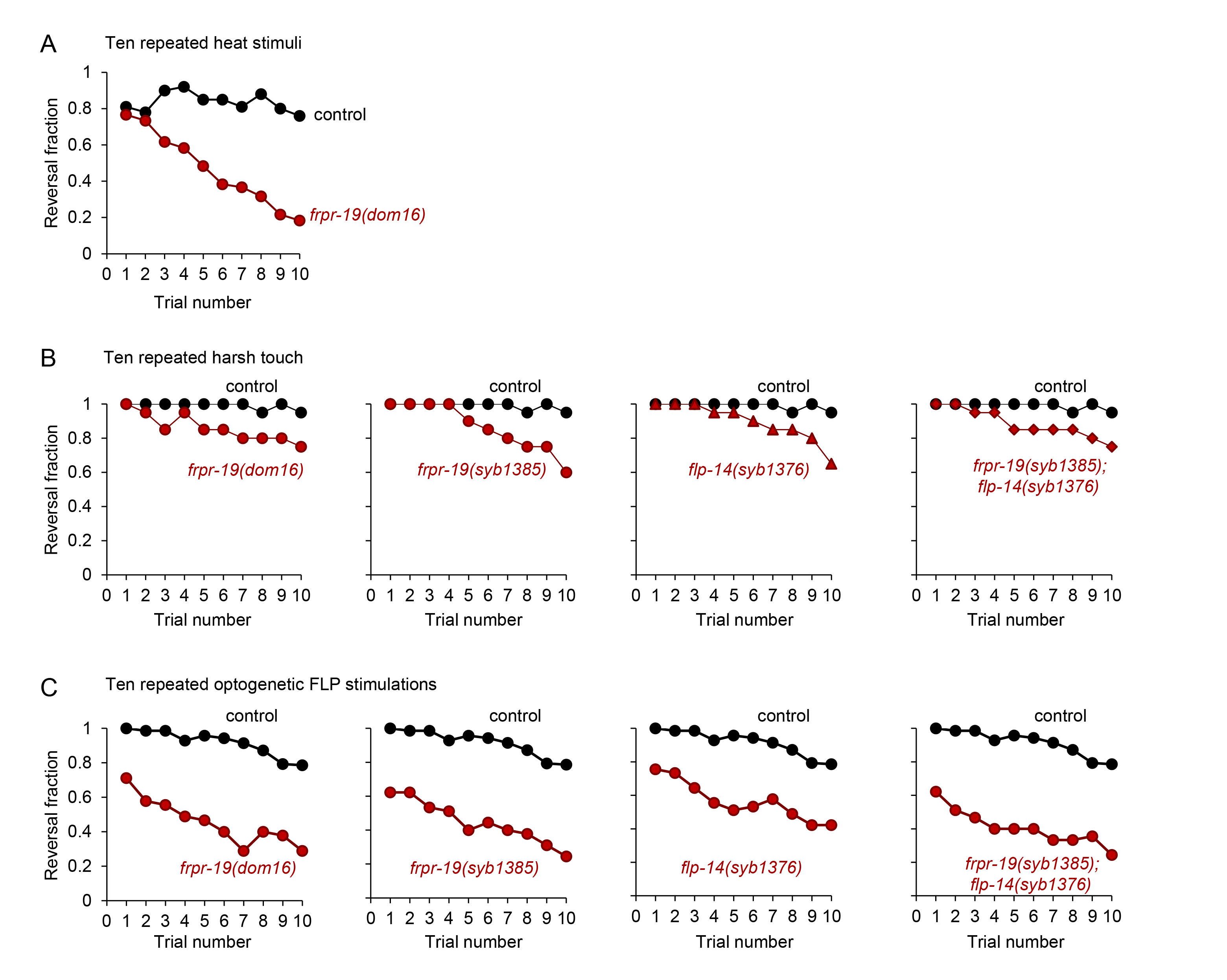

Supplement: S3 Fig — Fraction of stimuli producing a reversal response, scored as in Fig 5, but presented without normalizing to the maximal response. (A) Noxious heat stimuli. Data corresponding to Fig 5B. (B) Harsh touch stimuli. Data corresponding to Fig 5E and 5H. For harsh touch stimuli, the normalization had no impact because the maximal response was 100% for every genotype. Therefore, the harsh touch data series are identical in the two figures. (C) Optogenetic stimuli. Data corresponding to Fig 5J. (TIF) [file pgen.1009880.s003.tif]
