# Supplementary material for: Signaling via the FLP-14/FRPR-19 neuropeptide pathway sustains nociceptive response to repeated noxious stimuli in C. elegans
Source: PLoS Genet. 2021 Nov 8;17(11):e1009880. doi: 10.1371/journal.pgen.1009880 (PMC8601619; doi:10.1371/journal.pgen.1009880)
Supplement: S1 Table — (DOCX) [file pgen.1009880.s004.docx]

S1 Table. Strain list

| **Strain name** | **Genotype** | **Comment** |
| --- | --- | --- |
| N2 | Wild type | Wild type (WT) |
| DAG356 | *domIs355 [mec-3p*::*QF*, *mec-4p*::*QS*, *QUAS*::*CoChR*::*GFP*, *unc122p*::*RFP]* | [FLP::CoChR] FLP optogenetic background |
| DAG640 | *domIs355; frpr-19(dom16) IV* | New mutant (FRPR-19 Q389*) from the screen (*Marques et al.*, 2019 PMID:  31891575) in FLP optogenetic background, 4x backcrossed with the parental line DAG356 |
| DAG972-973 | *domIs355; frpr-19(dom16) IV*; *domEx972-973(F23F20 fosmid, unc122p::GFP)* | *frpr-19(dom16)* rescue with *frpr-19* fosmid *F23F20*. Fig 1C |
| DAG1248 | *domIs355; frpr-19(syb1397) IV* | FRPR-19 Q389* mutation made by genome editing (SunyBiotech, China) in FLP optogenetic background, 4x backcrossed with wild type. Fig 2 |
| DAG1249 | *domIs355; frpr-19(syb1385) IV* | 1323 bp deletion, removing 1-3 and creating out-of-frame transcripts (loss of *frpr-19a* and *frpr-19b*) in FLP optogenetic background, 4x backcrossed with wild type. Fig 2 |
| DAG1251 | *domIs355; frpr-19(syb1379) IV* | 890 bp deletion of alternative intron 6 (loss of *frpr-19a*) in FLP optogenetic background, 4x backcrossed with wild type. Fig 2 |
| DAG1250 | *domIs355; frpr-19(syb1392) IV* | 489 bp deletion removing alternative exon 7 (loss of *frpr-19b*) in FLP optogenetic background, 4x backcrossed with wild type. Fig 2. |
| DAG1388-1390 | *domIs355; domEx1388-1390 [frpr-19p::frpr-19GS::SL2::mCherry 20 ng/μl, unc-122p::GFP 20 ng/μl]* | *frpr-19* overexpression with *frpr-19* promoter driving *frpr-19* genomic sequence (GS). Fig 2C and 3D |
| DAG1198-1200 | *domIs355; domEx1198-1200 [frpr-19p::frpr-19aCDS::SL2::mCherry 20 ng/μl, frpr-19p::frpr-19bCDS::SL2::mCherry 20 ng/μl,unc-122p::GFP 20 ng/μl]* | *frpr-19A* and *frpr-19B* overexpression with *frpr-19* promoter driving *frpr-19a* and *b* coding sequences (CDS). Fig 2C |
| DAG1201-1203 | *domIs355; domEx1201-1203 [frpr-19p::frpr-19aCDS::SL2::mCherry 20 ng/μl,unc-122p::GFP 20 ng/μl]* | *frpr-19A* overexpression with *frpr-19* promoter driving *frpr-19a* coding sequence (CDS). Fig 2C |
| DAG1160-1162 | *domIs355; domEx1160-1162 [frpr-19p::frpr-19bCDS::SL2::mCherry 20 ng/μl,unc-122p::GFP 20 ng/μl]* | *frpr-19B* overexpression with *frpr-19* promoter driving *frpr-19b* coding sequence (CDS). Fig 2C |
| DAG1197 | *domEx1197 [frpr-19p::frpr-19aCDS::SL2::mCherry 20 ng/μl ; mec-3p::mNeongreen3xFLAG::unc-54UTR 20 ng/μl]* | *frpr-19* promoter reporter in red with FLP marker in green. Fig 3A |
| DAG1415-1416,1446 | *domIs355; frpr-19(syb1385) IV; domEx1415-1416,1446 [frpr-19p::frpr-19GS::SL2::mCherry 2 ng/μl, unc-122p::GFP 20 ng/μl]* | *frpr-19(syb1385)* rescue attempts with *frpr-19* promoter driving *frpr-19* genomic sequence (GS) (2 ng/μl). Fig 3C |
| DAG1412-1414 | *domIs355; frpr-19(syb1385) IV; domEx1412-1414 [mec-3p::frpr-19GS::SL2::mCherry 20 ng/μl, unc-122p::GFP 20 ng/μl]* | *frpr-19(syb1385*) rescue with *mec-3* promoter driving *frpr-19* genomic sequence (GS). Fig 3C |
| DAG1409-1411 | *domIs355; frpr-19(syb1385) IV; domEx1409-1411 [glr-1p::frpr-19GS::SL2::mCherry 20 ng/μl, unc-122p::GFP 20 ng/μl]* | *frpr-19(syb1385)* rescue with *glr-1* promoter driving *frpr-19* genomic sequence (GS). Fig 3C |
| DAG1379-1381 | *domIs355; frpr-19(syb1385) IV; domEx1379-1381 [frpr-19p::frpr-19GS::SL2::mCherry 20 ng/μl, unc-122p::GFP 20 ng/μl]* | *frpr-19(syb1385)* rescue attempt with *frpr-19* promoter driving *frpr-19* genomic sequence (GS) (20 ng/µl). S2 Fig |
| DAG1169,1173-1174 | *domIs355; frpr-19(syb1385) IV; domEx1169, 1173-1174 [frpr-19p::frpr-19ACDS::SL2mCherry 20 ng/μl ; frpr-19p::frpr-19BCDS::SL2mCherry 20 ng/μl ;unc122p::GFP 20 ng/μl]* | *frpr-19(syb1385)* rescue attempt with *frpr-19* promoter driving *frpr-19a* and *b* coding sequences (CDS) (20 ng/µl). S2 Fig |
| DAG1349-1350 | *domIs355; frpr-19(syb1385) IV; domEx1349-1350 [frpr-19p::frpr-19ACDS::SL2mCherry 2 ng/μl ; frpr-19p::frpr-19BCDS::SL2mCherry 2 ng/μl ;unc122p::GFP 20 ng/μl]* | *frpr-19(syb1385)* rescue attempt with *frpr-19* promoter driving *frpr-19a* and *b* coding sequences (CDS) (2 ng/µl). S2 Fig |
| DAG1385-1387 | *domIs355; domEx1385-1387 [mec-3p::frpr-19GS::SL2::mCherry 20 ng/μl, unc-122p::GFP 20 ng/μl]* | *frpr-19* overexpression with *mec-3* promoter driving *frpr-19* genomic sequence (GS) Fig 3D |
| DAG1382-1384 | *domIs355; domEx1382-1384 [glr-1p::frpr-19GS::SL2::mCherry 20 ng/μl, unc-122p::GFP 20 ng/μl]* | *frpr-19* overexpression with *glr-1* promoter driving *frpr-19* genomic sequence (GS) Fig 3D |
| PT501 | *flp-8(pk360) X* | 1414 bp deletion removing entire coding sequence, 6x backcrossed with wild type |
| DAG1149 | *domIs355; flp-8(pk360) X* | *flp-8(pk360)* in FLP optogenetic background. Fig 4 |
| DAG1252 | *domIs355; flp-14(syb1376) III* | 1254 bp deletion removing the coding sequence for the 4 peptides in FLP optogenetic background, 4x backcrossed with wild type. Fig 4 and 5 |
| DAG1255 | *domIs355; flp-8(pk360) X; flp-14(syb1376) III* | double-mutant in FLP optogenetic background. Fig 4 |
| DAG1254 | *domIs355; frpr-19(syb1385) IV; flp-14(syb1376) III* | double-mutant in FLP optogenetic background. Fig 4 and 5 |
| DAG1253 | *domIs355; frpr-19(syb1385) IV; flp-8(pk360) X* | double-mutant in FLP optogenetic background. Fig 4 |
| DAG1358-1360 | *domIs355; flp-14(syb1376) III; domEx1358-1360 [flp-14p::flp-14CDS::SL2::mCherry 20 ng/μl, unc-122p::GFP 20 ng/μl]* | *flp-14(syb1376)* rescue with *flp-14* promoter driving *flp-14* coding sequence (CDS). Fig 4 |
| DAG1484-1486 | *domIs355; flp-14(syb1376) III; domEx1484-1486 [mec-3p:: flp-14CDS::SL2::mCherry 20 ng/μl, unc-122p::GFP 20 ng/μl]* | *flp-14(syb1376)* rescue with *mec-3* promoter driving *flp-14* coding sequence (CDS). Fig 4 |
| DAG1481-1483 | *domIs355; flp-14(syb1376) III; domEx1481-1483[glr-1p:: flp-14CDS::SL2::mCherry 20 ng/μl, unc-122p::GFP 20 ng/μl]* | *flp-14(syb1376)* rescue with *glr-1* promoter driving *flp-14* coding sequence (CDS). Fig 4 |
